# Impairment in Task-Specific Modulation of Muscle Coordination Correlates with the Severity of Hand Impairment following Stroke

**DOI:** 10.1371/journal.pone.0068745

**Published:** 2013-07-16

**Authors:** Sang Wook Lee, Kristen Triandafilou, Blair A. Lock, Derek G. Kamper

**Affiliations:** 1 Department of Biomedical Engineering, Catholic University of America, Washington, DC, United States of America; 2 Center for Applied Biomechanics and Rehabilitation Research, MedStar National Rehabilitation Hospital, Washington, DC, United States of America; 3 Sensory Motor Performance Program, Rehabilitation Institute of Chicago, Chicago, Illinois, United States of America; 4 Center for Bionic Medicine, Rehabilitation Institute of Chicago, Chicago, Illinois, United States of America; 5 Department of Biomedical Engineering, Illinois Institute of Technology, Chicago, Illinois, United States of America; The University of Queensland, Australia

## Abstract

Significant functional impairment of the hand is commonly observed in stroke survivors. Our previous studies suggested that the inability to modulate muscle coordination patterns according to task requirements may be substantial after stroke, but these limitations have not been examined directly. In this study, we aimed to characterize post-stroke impairment in the ability to modulate muscle coordination patterns across tasks and its correlation with hand impairment. Fourteen stroke survivors, divided into a group with severe hand impairment (8 subjects) and a group with moderate hand impairment (6 subjects) according to their clinical functionality score, participated in the experiment. Another four neurologically intact subjects participated in the experiment to serve as a point of comparison. Activation patterns of nine hand and wrist muscles were recorded using surface electromyography while the subjects performed six isometric tasks. Patterns of covariation in muscle activations across tasks, i.e., muscle modules, were extracted from the muscle activation data. Our results showed that the degree of reduction in the inter-task separation of the multi-muscle activation patterns was indicative of the clinical functionality score of the subjects (mean value = 26.2 for severely impaired subjects, 38.1 for moderately impaired subjects). The values for moderately impaired subjects were much closer to those of the impaired subjects (mean value = 46.1). The number of muscle modules extracted from the muscle activation patterns of a subject across six tasks, which represents the degree of motor complexity, was found to be correlated with the clinical functionality score (*R* = 0.68). Greater impairment was also associated with a change in the muscle module patterns themselves, with greater muscle coactivation. A substantial reduction in the degrees-of-freedom of the multi-muscle coordination post-stroke was apparent, and the extent of the reduction, assessed by the stated metrics, was strongly associated with the level of clinical impairment.

## Introduction

Stroke often has a dramatic impact on upper extremity motor control [1], especially in the hand [2]. Among the resulting deficits noted, profound voluntary weakness [3]–[5], reduced active range of motion [6]–[8], and misdirected endpoint forces [9], [10] are especially striking. These impairments can substantially reduce functional use of the upper limb.

While tissue alterations such as contracture formation [11], increased passive joint impedance [12], and changes in muscle fiber type [13] can contribute to the observed deficits, the fundamental impairment mechanisms appear to be neurological in origin. In the hand, at least, the major sources of impairment arise from motoneuronal hyperexcitability, reduced voluntary activation of motoneurons, and altered activation patterns. The first two sources have been widely described. For example, motoneuronal hyperexcitability [14], as evidenced by spasticity [15], has been well characterized [11], [16], [17]. This hyperexcitability is also thought to lead to excessive coactivation of certain muscles [18], [19] and difficulty in terminating muscle activity once initiated [20]. Diminished amplitude of voluntary muscle activation is present and contributes to weakness. For example, motor unit firing rates have been shown to be compressed [21], [22], thereby decreasing the strength of muscle contraction. The ability to voluntarily recruit motor units fully also appears to be diminished after stroke [23]–[25].

The nature of the muscle activation patterns during functional task performance, however, has not been examined to an equivalent extent. While increased inter-joint coupling during upper extremity motor tasks has been shown [6], [26]–[33] and has been attributed to difficulty with minimizing activation coupling among specific muscles such as shoulder abductors and elbow flexors [34], [35], the capacity of stroke survivors to modulate activation patterns with task, specifically for hand muscles, is not well known. In the hand, we have noted that stroke survivors experience difficulty in to controlling the activation of individual muscles [5]. The normalized activation range of a given muscle is greatly restricted such that the activation level changes relatively little from task to task, in contrast to what is observed in neurologically intact individuals.

Therefore, potential impairment in the ability of stroke survivors to create and modulate muscle activation patterns across tasks could be one of the main contributors to functional degradation of the hand. Most manual tasks require a complex spatiotemporal coordination of multiple joints [36], in which the sophisticated biomechanical system of the hand transforms muscle forces to the joint movements/torques in order to achieve the task goals [37]–[41]. More importantly, since a variety of grip patterns that require different kinematics and kinetics are typically associated with functional use of the hand [42], [43], not only is an accurate coordination of many muscles necessary to perform each manual task [36], [44]–[46], but proper modulation of muscle activation patterns across multiple tasks (e.g., [47], [48]) is also crucial in order to provide the versatility which makes the human hand so dexterous.

After stroke, the capability to create and modulate muscle activation patterns is thought to be compromised, but multi-muscle coordination of stroke survivors and its modulation across distinct manual tasks has not been probed directly. Higher-order deficits found to emerge following stroke, such as diminished anticipatory control of the hand [49] and arm [50] and the development of compensatory motor control strategies [51], may arise from reductions in the available muscle activation workspace [52]. Each region of this workspace corresponds to a specific spatial coordination pattern of the muscles required to perform a task, which should be distinctly separated from other regions responsible for other tasks.

Accordingly, this study aims to directly examine the ability of stroke survivors to access different regions of the muscle activation workspace to perform a variety of tasks. In order to attain this goal, muscle activation patterns of stroke survivors and subjects with no impairment were examined during performance of different isometric manual tasks. First, in order to provide a quantitative measure of difference in the muscle coordination patterns across tasks, a Squared Euclidean Distance (SED) matrix that quantifies inter- and intra-task variability in multi-muscle coordination patterns of each subject was computed for each subject. Then, patterns of covariation in muscle activation (i.e., muscle modules) across tasks were extracted from the activation patterns in order to assess the extent to which each subject could utilize the potential workspace of muscle activations. Note that, in contrast to previous studies that examined modular structure of stroke survivors by extracting muscle modules to explain limitation in a temporal coordination of muscle activation during a single task (i.e., walking [53] or reaching [54], [55]) or impairment in modulation of the muscle coordination during tasks of similar nature (i.e., multi-directional isometric force generation with the arm [56], [57]), our study focused on the *variability in the modulation of multi-muscle coordination across functional tasks of a distinct nature*, such as between keeping the hand fully open and creating a power grasp. We hypothesized that diminished capacity to explore the workspace across tasks would correlate with the level of overall clinically assessed impairment. This result would suggest that this inability to create and appropriately modulate muscle activation patterns according to manual task requirements also greatly contributes to the functional deficits of stroke survivors. Lastly, we estimated correlation coefficients of activation levels of all muscle pairs across tasks, which enabled us to identify specific muscle pairs with a high degree of coupling/co-variation.

## Materials and Methods

### Ethics Statement

The experimental protocol was approved by the Northwestern University Institutional Review Board, and written informed consent was obtained from each subject.

### Subject Characteristics

Fourteen subjects with chronic hemiparesis (ages 41–81 years, mean ± SD age: 58±10 years; 10 males and 4 females; minimum 2 years since the onset of stroke) participated in the study. Subjects with subcortical or multiple strokes were excluded. Subjects were selected based on hand motor impairment level, as assessed by a research occupational therapist. Impairment was classified on an ordinal scale from 1 (most severe impairment – flaccid paralysis) to 7 (able to perform all tasks on scale) in accordance with the Stage of Hand component of the Chedoke-McMaster Stroke Assessment scale [58]. Subjects were categorized into two groups according to their Chedoke-McMaster Stage of Hand (CMSH): a subject group with severe hand impairment (CMSH 2–3; Subjects 1–8; 8 subjects; mean ± SD age: 56±6 years), and a subject group with moderate hand impairment (CMSH 4–5; Subjects 9–14; 6 subjects; mean ± SD age: 61±14 years). Four neurologically intact subjects (Subjects 15–18; 4 subjects; ages 24–35 years, mean ± SD age: 28±5 years) were also recruited as an unimpaired group to provide comparison.

### Experimental Protocol

Nine pairs of disposable, self-adhesive silver/sliver chloride surface electrodes (Myosystem 1400A, Noraxon, AZ, USA) were used to record EMG signals from the following nine muscles/muscle groups of each subject: thenar muscle group (THE), first dorsal interosseous (FDI), hypothenar muscle group (HTH), flexor digitorum superficialis (FDS), extensor digitorum communis (EDC), flexor carpi radialis (FCR), flexor carpi ulnaris (FCU), extensor carpi radialis (ECR), and extensor carpi ulnaris (ECU). In order to ensure the accurate placement of each electrode, EMG signals from the target muscle and its adjacent muscles were inspected simultaneously while subjects performed several hand/wrist movements associated with these muscles after the placement. If cross-talk between channels was observed, the electrodes were repositioned. Specifically, for the extrinsic hand muscles (i.e., EDC and FDS), we targeted the first and second compartments of these muscles, and these electrodes were placed on the distal portion of the muscles in order to minimize potential signal cross-talk with the wrist muscles (i.e., FCR/U and ECR/U).

Before the experiment started, subjects first performed a series of maximum isometric contractions in order to obtain the maximum level of EMG activity for the muscles of interest. Namely, subjects performed maximum thumb flexion/abduction, finger extension/flexion, finger abduction/adduction, and wrist extension/flexion, each of which lasted approximately 3 seconds. During the experiment, the EMG signals were amplified and then bandpass filtered between 20 and 500 Hz, prior to sampling at 1000 Hz.

**Figure 1:**
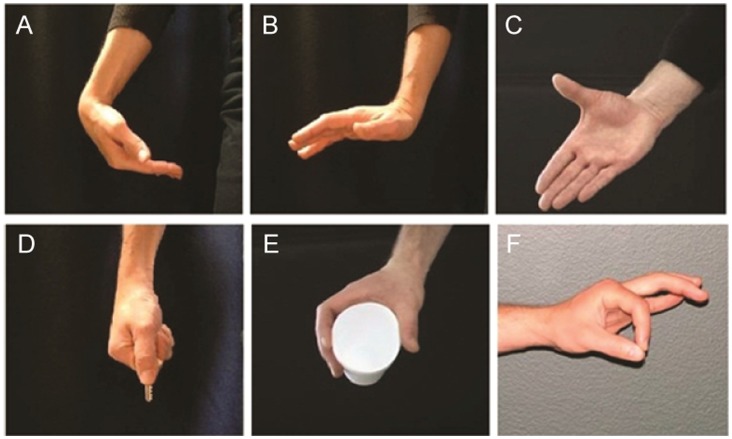
Target isometric tasks: (A) wrist flexion (WF), (B) wrist extension (WE), (C) finger extension (FE); hand open, (D) lateral pinch (LP), (E) power grip (PG), and (F) tip pinch (TP).

Subjects were instructed to perform six isometric tasks chosen to encourage exploration of the muscle activation workspace: wrist flexion (WF), wrist extension (WE), finger extension (“hand open”; FE), lateral pinch (LP), power grip (PG), and tip pinch (TP) (Fig. 1). Subjects were seated in front of a table on which they rested their arm in order to minimize the effects of potential distal-proximal coupling (i.e., flexor/extensor synergy; [26]) on hand muscle activation. The height of the chair was adjusted for each subject, and he/she was asked to adjust the hand location in order to attain elbow flexion of about 90° and shoulder flexion of about 30°. Subjects were allowed to change the location of their hand in case they had difficulty achieving the desired posture due to limited range of motion of their more proximal arm joints (i.e., elbow and shoulder). The positions of the wrist and fingers/thumb were not constrained during task performance, as each subject adopted certain ‘subject-specific’ compensatory strategies to facilitate the task performance (e.g., [51]).

The experiment consisted of eight blocks of trials, separated by 5-minute rest periods. In each block, subjects performed two 3-second isometric contractions for each task. Thus, each task was repeated 16 times. The order of the tasks within each block was randomized. A relaxation/preparation period of 15–20 seconds was instituted between consecutive tasks to allow for a return to the resting state and for preparation for the next task. During these periods, subjects were instructed to pre-shape their hands in order to prepare for the next target task so that only isometric contraction of the muscles was recorded during the target task performance. During task performance, subjects were asked to actively produce constant force (at a moderate level of exertion) specific to each task. For the hand open and wrist extension/flexion tasks, they contracted against passive tissue resistance at the end of their active ranges of motion. For the three grip tasks, subjects were asked to contract against physical objects, i.e., a cylindrical object during the power grip task and a key during the tip pinch and the lateral pinch tasks. The objects were placed in the hand of each subject by the experimenter.

Note that the purpose of the experiment was to examine the variation in muscle activation patterns that each subject employed across tasks when they ‘attempted’ to perform the given tasks. Therefore, subjects were encouraged to attempt to perform the tasks even in cases where they were not able to physically achieve the task due to their impairment. Actual task performance was not monitored; we expected task performance to vary across subjects. While subjects gave verbal confirmation of their moderate level of effort, it should be noted that past studies have shown that the nature of the activation patterns for isometric hand tasks do not change with level of force [45], [59]. The overall amplitude changes, but the pattern does not, so even if the level of effort varied from subject to subject, this would not significantly affect our results.

**Figure 2:**
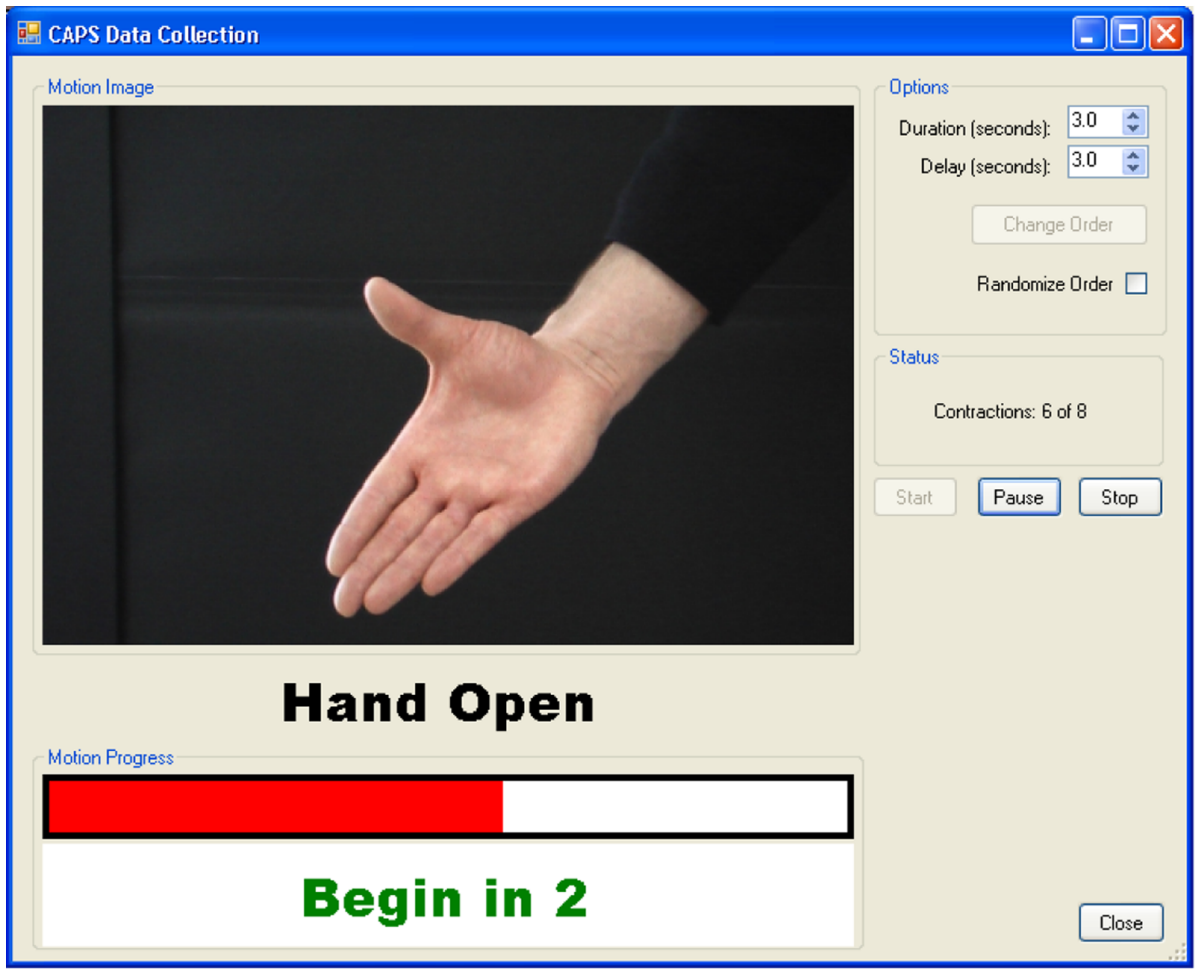
Visual feedback regarding the target task and task initiation/termination time provided to subjects.

Specialized graphical user interface (GUI) software (CAPS, version 2.4, Center for Bionic Medicine, Rehabilitation Institute of Chicago, Chicago, IL) was used to guide subjects throughout the experiment (Fig. 2). This software provided each subject with visual feedback regarding the task and its initiation/termination time, and recorded EMG signals during each task.

### Data Analysis

We have utilized three methods to examine the impairment in task-specific modulation of multi-muscle coordination: 1) estimation of Euclidean distance between muscle coordination patterns that simply quantifies the magnitude of between-task discrepancy; 2) muscle module extraction that can capture structural similarity between the muscle coordination patterns; and 3) correlation analysis that assesses the degree of coactivation for each individual muscle pair and its visualization.

#### EMG processing: Estimating muscle activation level

Each EMG signal was processed with a 60-Hz notch-filter to remove noise. The root-mean-square (RMS) value of each signal for the entire 3-second duration of the trial was then calculated in order to represent the activity of the muscle. This quantity was normalized by the RMS value of the maximum voluntary contraction, produced on the same electrode during the maximal activation trials. Here, ulnar and radial wrist extensor muscle activations (ECR and ECU) and flexor muscle activations (FCR and FCU) were each averaged to represent overall wrist extensor (ECR/U) and flexor (FCR/U) activities, respectively.

#### Inter-task separation and intra-task variability in muscle activation: Euclidean distance

In order to quantify the inter-task separation of the muscle activation patterns of the subjects, the squared Euclidean distance (SED) matrix between the normalized EMG vectors of the six target tasks was calculated for each subject. 
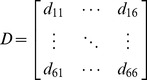
(1)where
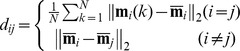
(*i*, *j* = 1: WF, 2: WE, 3: FE, 4: LP, 5: PG, 6: PP; *k* = 1, …, 16; 2 task performances/block × 8 blocks)

Here, **m** *_i_*(*k*) is the 7-element normalized EMG vector of task *i* at *k^th^* task performance:
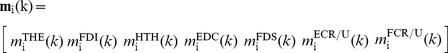
and denotes the mean EMG vector (i.e. average muscle activation levels) of task *i*:
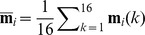



The *i^th^* diagonal element of the SED matrix d_ii_ corresponds to the intra-task (or within-task) variability of task *i*, whereas the off-diagonal element d_ij_ denotes the inter-task separation between task *i* and task *j*. Note that d_ii_ is derived from the definitions of within-group mean-square statistics used in MANOVA, and d_ij_ denotes the group difference used in *post hoc* Tukey’s honestly significant difference (HSD) test [60]. Elements of the SED matrix can thus be employed to determine which groups differ from each other (i.e., to identify significant difference in activation patterns between two tasks). Greater inter-task separation d_ij_ denotes better task-dependent modulation, while greater intra-task variability d_ii_ implies a less consistent muscle activation pattern for the given task *i*. The SED matrix provides quantitative measures of inter-task separation and intra-task variability in muscle activation patterns [61].

In order to assess the ability of stroke survivors to create and modulate multi-muscle activation patterns across tasks, the inter-task separation and the intra-task variability were quantified and compared via statistical analyses. Namely, in order to test if the inter-task separation of the activation pattern (i.e., between-task separation) was greater than its intra-task variation (i.e., within-task variability), a one-way MANOVA was performed for both groups (i.e., severely impaired and moderately impaired), with the normalized EMG vector as a dependent variable and the task (6 levels) as an independent variable (PASW Statistics 18; SPSS Inc., Chicago, IL). A significance level was set to 0.05. *Post hoc* Tukey’s HSD tests were performed to examine differences between tasks (_6_C_2_ = 15 task-pairs).

#### Muscle module extraction

Although SED values provide quantitative measures for the inter-task separation of muscle activation patterns, such metrics may not be appropriate to capture ‘structural complexity’ in multi-muscle activation, i.e., spatial coordination patterns of multiple muscle activation levels, as SED values can simply calculate the difference (i.e., distance) between two activation vectors but cannot recognize similarity between these vectors (i.e., similar direction but different magnitude). Therefore, we extracted muscle modules, a coordinated action of a group of muscles with specific task-dependent profile, from the muscle activation patterns. Modular decomposition of muscle activation has been widely used to elucidate organizational structure of motor control (for example, [62]–[64]).

In this study, a muscle module is defined as ‘a group of muscles activated in synchrony with fixed relative gains [65]’, which corresponds to consistent ‘spatial’ characteristics of muscle activation patterns during isometric tasks. For each subject, a nonnegative matrix factorization (NMF) was employed to extract functional muscle modules [66]. For each task *i*, the muscle activation (EMG) vector of the *k* ^th^ trial was represented using a weighted sum of functional muscle modules;
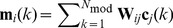
(2)


(*i* = 1, 2, …, 5: task; *j* = 1, 2, …, N_mod_: module; *k* = 1, 2, …, 16: trial)

Here **W** *_ij_* is a nonnegative coefficient that defines how the module activation varies as a function of task, **c** *_j_* defines the relative activation level (between 0 and 1) of each muscle within the module, and N*_mod_* the number of muscle modules. Note that our muscle module structure is different from previous studies that examined temporal coordination of multiple muscles during a single task (i.e., walking [53] or reaching [54], [55]). Rather, we employed a definition of muscle module combination similar to the previous studies that examined muscle modules in postural tasks [63], [65]; the directional tuning of muscle modules (in response to disturbance) in these previous studies was replaced by the task-dependent tuning in this study.

The number of modules required to best characterize the data was determined by the total variance accounted for (VAF), which is defined as 100 × the coefficient of determination from the uncentered Pearson correlation coefficient [63]. We also used a local criterion to add muscle modules in order to account for the task-specific modulation of a particular muscle, i.e., VAF of each muscle (local VAF), which may not be considered by the global criterion (total VAF) (e.g., [65]). In an iterative procedure, the number of muscle modules was increased from one to six, and W_ij_ and c_j_ were obtained through an optimization procedure using the *nnmf* function of the Statistics Toolbox in MATLAB (MathWorks, Inc., Natick, MA), which factors the non-negative matrix of muscle activations into non-negative factors W (task-dependent tuning curves) and C (modules). A minimum of twenty optimizations were performed with varying initial conditions to avoid being trapped in local minima. For each subject, the minimum number of modules that resulted in total VAF >90% and all local VAFs >75% was determined [65].

Once the muscle modules were extracted from all subjects, similarity between the modules across subjects was evaluated. In order to quantify the degree of similarity of the muscle modules between subjects, a correlation coefficient (*R*), or a scalar-product similarity between the modules was estimated. Similar measures have been used in other studies (e.g., [65], [67]). Between two subjects, *R*-values were first estimated for all possible module pair combinations, and the module pairs were then determined in the way that the highest average *R*-values were resulted. First, for the stroke subjects, *R*-values were first estimated between the modules from subjects with the same number of muscle modules. Then the *R*-values were estimated between subjects with different number of modules. The similarity in muscle modules across control subjects were estimated in a similar way. Then the similarity in the modules from stroke survivors and those from control subjects were estimated. To evaluate the chance level of the similarity between the muscle modules, we first generated two sets of 1,000 random muscle modules of the same length (*n* = 7) using the mean and standard deviation of the EMG dataset (normrnd function in MATLAB). Then, we estimated the similarity (*R*-value) of all possible pairs of the random modules (1,000 × 1,000 = 1,000,000 pairs in total), and the 95^th^ percentile of the distribution of these random similarity measures (*R = *0.68) was set to our threshold *R*-value. Note that similar analyses were performed in previous studies to determine the critical *R-*value [57], [64], [68]. We compared the actual similarity *R*-value with this critical *R-*value to examine statistical significance.

We also evaluated the similarity in the muscle modules across the three subject groups, i.e., severely-impaired subjects, moderately-impaired subjects, and unimpaired subjects, by calculating the VAF obtained by fitting muscle modules estimated from one subject group to the muscle activation patterns of another subject group (i.e., cross-validation). The VAF values reflect the quality of the global reconstruction of a given muscle coordination patterns across tasks. Here, to compare the estimated VAF to the chance level of the VAF value, we fit the aforementioned random muscle modules, generated from the mean and standard deviation of the actual EMG dataset, to the original EMG data [57]. This procedure was repeated 100 times to obtain an empirical distribution of the VAF values. Then, the 95 percentile of the VAF value was used as a critical VAF value (VAF_crit_).

Finally, we also tested if the muscle modules of stroke survivors can be reconstructed by merging those of unimpaired subjects, as shown in previous studies [55]. Briefly, the muscle modules of each stroke survivor were modeled as a linear combination of the muscle modules of unimpaired subjects. The coefficients determining the contribution of muscle modules of unimpaired subjects were estimated using the *lsqnonneg* function in MATLAB.

#### Assessment of the degree of coactivation of muscle pairs

In addition, in order to identify specific muscle pairs with higher degrees of coupling (i.e., increased level of coactivation across tasks), a correlation analysis was performed in order to assess the degree of coactivation of different muscle pairs during manual tasks and wrist isometric tasks. For each subject, a 7×7 correlation matrix was constructed, with each element (*i*, *j*) equal to the correlation coefficient *R* between the *i^th^* and *j^th^* muscle activation levels across all six tasks. Each off-diagonal element denotes the degree of covariation of two muscles (or muscle groups) across tasks. We calculated 95% bootstrap confidence intervals for the correlation coefficients (*bootstrp* function; MATLAB Statistics Toolbox, Natick, MA) in order to identify statistically meaningful correlations (i.e., *R*-values different from zero).

We also constructed two graphs that visualize the portion of muscle activation workspace that is utilized by each subject during the task performance. In the first graph, each axis denotes the activation level of one of the three intrinsic hand muscles, i.e., FDI, THE, and HTH. The second graph depicts the muscle workspace of the three extrinsic hand muscles, i.e., FDS, EDC, and FCR/U. These plots graphically demonstrate what portion of the entire muscle workspace is utilized during the task performance.

## Results

### Modulation of Muscle Activation Pattern with Task: Inter-task vs. Intra-task Variability

Quantified inter-task separation in multi-muscle activation patterns of stroke survivors was indicative of their impairment level (Table 1, Table 2, Table 3). Estimated squared Euclidean distance (SED) matrices showed that the inter-task separation of muscle activation vectors of severely impaired subjects (i.e., magnitudes of the off-diagonal elements in the SED matrix) (mean value = 26.2; Table 1) was smaller than that of moderately impaired subjects (mean value = 38.1; Table 2). This value for moderately impaired subjects was closer to the inter-task separation observed in unimpaired subjects (mean value = 46.1; Table 3). The larger separation indicates greater modulation of activation pattern with task. Conversely, the intra-task variability of the no-impairment subjects was lower than that of stroke survivors (i.e., smaller diagonal element magnitudes in their SED matrices), indicating that the activation patterns for a given task were more consistent for the neurologically intact subjects. For all three groups, however, inter-task variability was still greater than intra-task variability (*p*<0.05).

**Table 1:** Mean Squared Euclidean Distance (SED) values of the muscle activation levels (%): severely-impaired subjects (CMSH 2–3).

		Task					
		WE	WF	FE	LP	PG	TP
**Task**	WE	23.2	27.7	22.5	31.2	48.3	34.0
	WF		25.0	19.5	29.9	41.7	32.7
	FE			17.1	21.4	48.9	25.9
	LP				24.3	35.4	8.1
	PG					37.3	31.7
	TP						25.9

* Diagonal elements denote intra-task variability.

**Table 2:** Mean Squared Euclidean Distance (SED) values of the muscle activation levels (%): moderately-impaired subjects (CMSH 4–5).

		Task					
		WE	WF	FE	LP	PG	TP
**Task**	WE	23.0	54.3	54.3	45.7	58.3	45.7
	WF		28.4	34.8	45.8	53.9	40.2
	FE			24.6	53.0	56.4	47.0
	LP				23.8	41.3	18.8
	PG					24.9	40.0
	TP						22.9

* Diagonal elements denote intra-task variability.

**Table 3:** Mean Squared Euclidean Distance (SED) values of the muscle activation levels (%): subjects with no impairment (CMSH 7).

		Task					
		WE	WF	FE	LP	PG	TP
**Task**	WE	18.9	41.1	48.0	71.2	54.2	59.8
	WF		12.1	41.5	81.3	57.9	64.6
	FE			17.0	73.6	51.8	60.7
	LP				17.1	51.9	33.6
	PG					19.8	42.1
	TP						18.4

* Diagonal elements denote intra-task variability.

**Figure 3:**
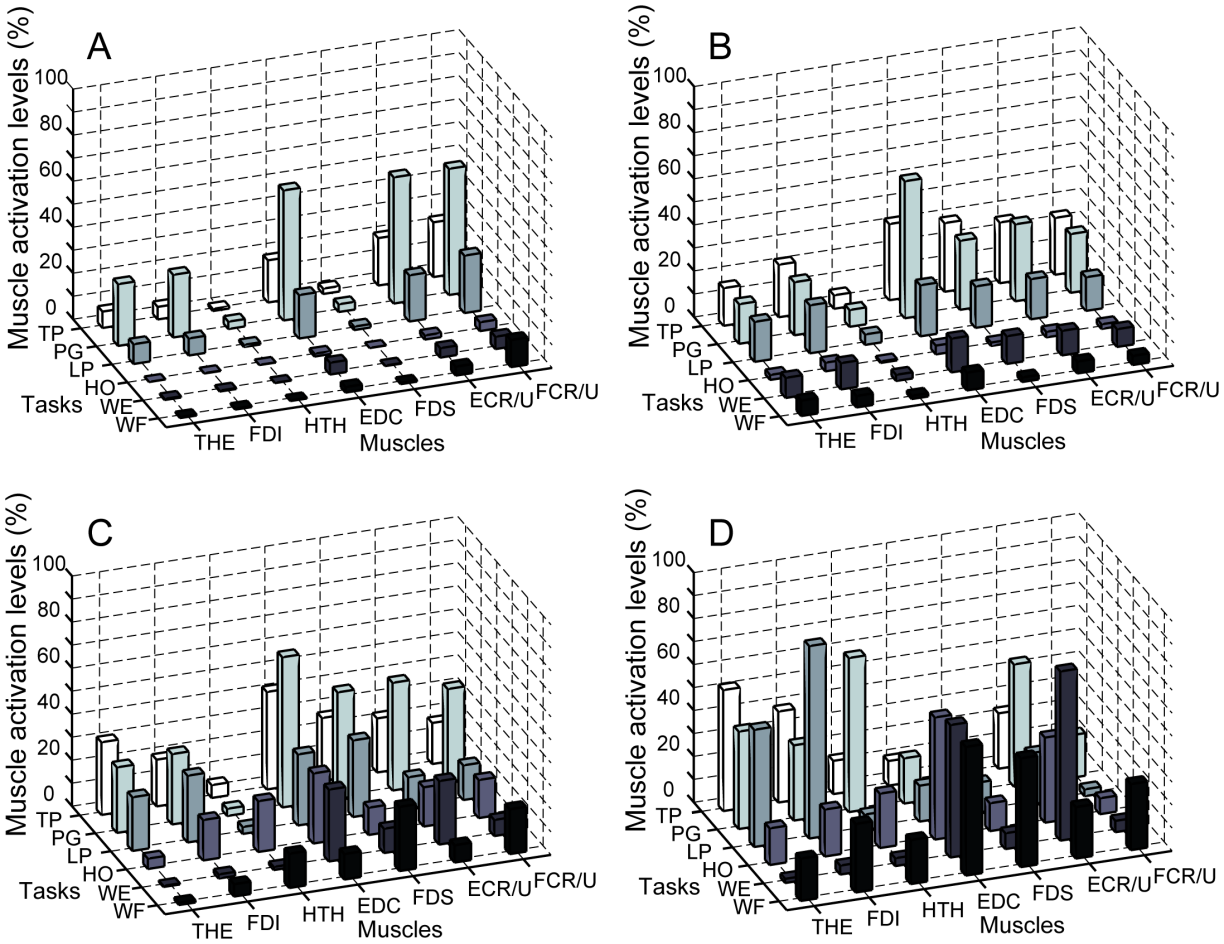
Muscle activation pattern of (A,B) severely impaired subjects: (A) Subject 3 (CMSH 3), (B) Subject 6 (CMSH 2); (C) moderately impaired subject: Subject 13 (CMSH 5); and (D) Unimpaired subject: Subject 16 (CMSH 7) across six tasks. Activation patterns of severely impaired subjects were very similar across different tasks (except overall magnitude) (A,B); qualitative examination of the data shows the inter-task variability, or complexity in muscle activation patterns across tasks, was greater in subjects with less impairment (i.e., higher CMSH).

Although MANOVA tests showed that muscle activation patterns across tasks are significantly different within each of the three subject groups, *post hoc* Tukey HSD tests with pairwise comparison (_6_C_2_ = 15 task-pairs) revealed that the activation levels of most muscles of severely impaired subjects were not statistically different between most task-pairs. In other words, muscle activation was not modulated with task for these subjects. Out of a total of 105 comparisons (15 task-pairs × 7 muscles), 70.4% (74 out of 105) demonstrated no significant difference (*p*>0.05) for the severely impaired subject group. Specifically, flexor digitorum superficialies (FDS) activity demonstrated no statistically significant difference between any task-pair for this group. Thenar (THE) muscle activity showed statistically significant difference only between tasks 3 and 5, i.e., finger extension vs. power grip. In contrast, first dorsal interosseous (FDI) and extensor carpi radialis/ulnaris (ECR/U) were the muscles that showed the most discrimination between the tasks, as significantly different muscle activation patterns were observed for nine and seven task-pairs, respectively. The total number of task pairs with no statistical difference was only 35 out of 105 pairs (33.3%) for moderately impaired subjects, thereby indicating greater modulation of individual muscles between tasks for these subjects. This latter value was similar to that observed in the subjects with no impairment (30 out of 105 pairs or 28.5%),

Qualitative evaluation of the muscle activation patterns of stroke survivors revealed that the degree of task-dependent adaptation of muscle activation patterns, or difference in muscle activation patterns across tasks, was generally lower in severely impaired subjects (Fig. 3A and B); in particular, many subjects from this group employed very similar activation patterns (‘subject-specific stereotypical patterns’) to perform tasks of very different natures. In many cases, changes in activation across tasks consisted largely of increases or decreases in overall muscle activation level of the same basic activation pattern, i.e., the same EMG vector, suggesting reduced dimensionality in their motor control patterns. Notably, the activation levels of all muscles of stroke survivors were higher during the three grip tasks than during other tasks. Differences in muscle activation patterns across tasks were more distinct in the moderately impaired subject group (Fig. 3C), as were the differences for the subjects with no impairment (Fig. 3D).

### Structural Dimensionality Reduction in Motor Control Post Stroke

**Figure 4:**
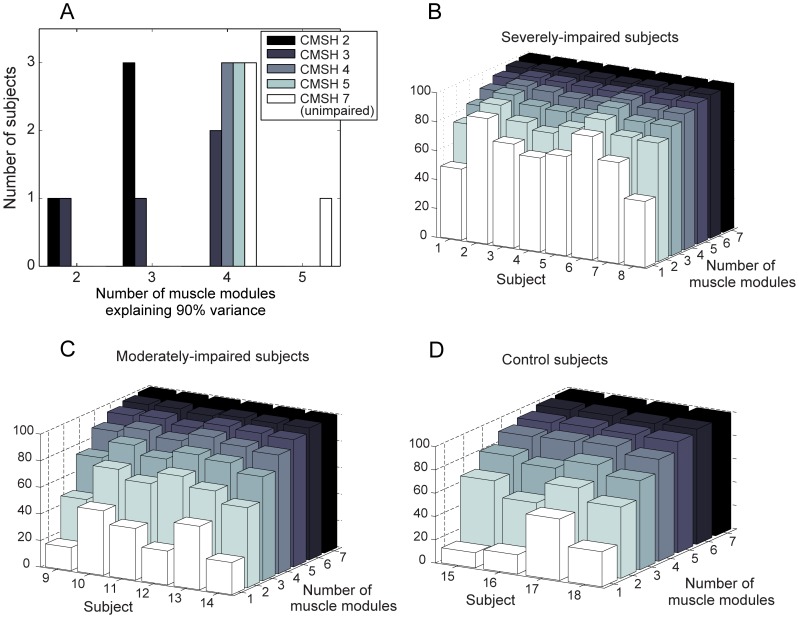
Dimensionality reduction from the muscle modules: (A) Number of modules required to explain more than 90% of the variance in the entire muscle activation patterns and 75% of the variance in each muscle activation pattern. Fewer modules were required to reconstruct the muscle activation patterns of more impaired subjects (lower CMSH). (B–D) Number of modules that explained 90% of the variance in the data for the three subject groups. For most severely impaired subjects (b: 6 out of 8), the first module explains more than 60% of the VAF, whereas the first module explains less than 35% of VAF for most moderately impaired subjects (C: 5 out of 6), and less than 30% of VAF for most subjects with no impairment (D: 3 out of 4).

Structural dimensionality reduction in the task-specific modulation of multi-muscle activation was captured by the muscle module extraction. The more impaired the stroke subject, the fewer modules were required to explain the variability in the muscle activation patterns across tasks (Fig. 4A). For the severely-impaired subjects, i.e., CMSH 2–3, two to four modules were required to reconstruct muscle activation patterns of all tasks (two modules - Subjects 2, 6; three modules - Subjects 3, 5, 7, 8; four modules – Subjects 1, 4; Fig. 4B). For the moderately impaired subjects (CMSH 4–5), four modules were required to reconstruct their muscle activation patterns (Subjects 9–14; Fig. 4C). In subjects with no impairment, at least four independent modules were required to reproduce muscle activation patterns across all six tasks; one subjects (subject 18) required five modules in order to reach 90% of the variance-accounted-for (VAF).

A positive correlation between the functional impairment score (i.e., CMSH) and the number of modules that account for the variability in the muscle activation patterns – which signifies the degree of motor complexity in multi-muscle activation – was observed, yielding the correlation coefficient (*R*) of 0.68.

In the severely impaired subject group, one muscle module (i.e., one stereotypical multi-muscle activation pattern) usually explained more than 60% of the overall variance (mean VAF by one module = 67.8%; Fig. 4B), while the VAF value by one module is approximately 35% for moderately impaired subjects (mean VAF by one module = 35.6%; Fig. 4C); it was less than 30% for subjects with no impairment (mean VAF by one module = 28.0%; Fig. 4D). Thus, the VAF by one module was negatively correlated with the subject impairment level (*R* = –0.71).

#### Muscle module composition: effect of functional impairment

**Figure 5:**
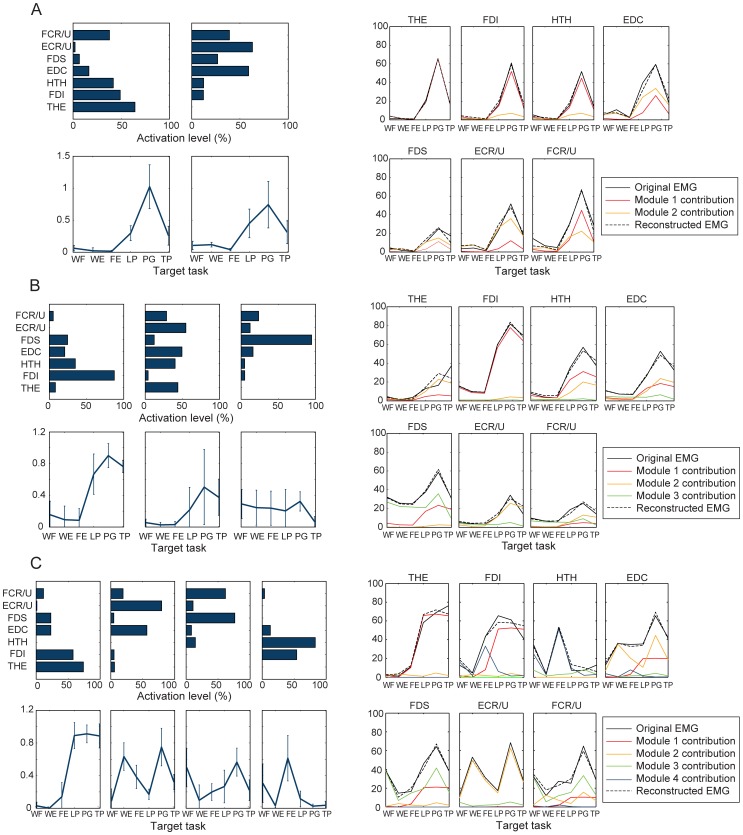
Muscle module – reconstructed activation pattern: (A) Severely impaired subject (Subject 2, low-complexity: 2 modules) (B) Severely impaired subject (Subject 3, medium-complexity: 3 modules) (C) Moderately impaired subject (Subject 13, high-complexity: 4 modules).

For stroke survivors, specifically for severely-impaired subjects, most muscle modules contained dominant components of more than three muscles, and each module was likely to be employed during multiple tasks (Fig. 5). The composition of the muscle modules of stroke subjects substantially varies across subjects.

**Figure 6:**
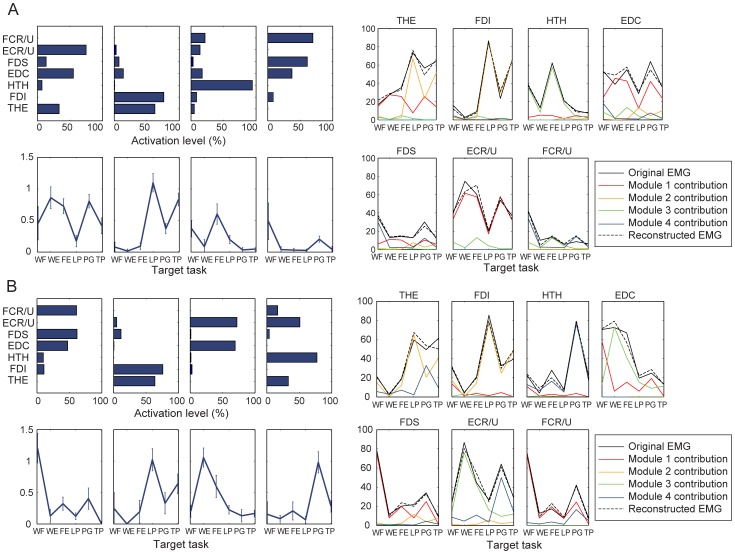
Muscle module – reconstructed activation pattern of subjects with no impairment: (A) Subject 15, (B) Subject 16. Note that a subset of the muscle modules (**c**_j_) and their tuning curves (**W**_ij_) were similar between these two subjects (i.e., module 2 of both subjects, module 1 of subject 15 and module 3 of subject 16, and module 4 of subject 15 and module 1 of subject 16).

In contrast, in subjects with no impairment, similar muscle modules (**c**_j_) and their tuning curves (**W**_ij_) were present across subjects (Fig. 6). Generally, for each subject, each module was responsible for one or two specific tasks.

The similarity analysis identified three common modules present across subjects according to the scalar-product similarity, or the correlation coefficient (*R*) value. Some differences did exist between the components of each module across subjects, but the composition of three modules was found to be fairly consistent across the unimpaired subjects. For instance, subjects 15 and 16 had four modules (Fig. 6A and B). The most prevalent module (module 2 for both subjects) consisted largely of thenar and FDI muscle activations; it was active primarily during the grip tasks, and the mean (SD) value of the correlation coefficients across all unimpaired subjects was 0.85 (0.12). The second prevalent module (module 1 in subject 15; module 3 in subject 16), consisting of EDC and ECR/U, was mainly active during wrist extension and finger extension, with the mean (SD) *R*-value of 0.81 (0.15) across subjects. The third module (module 4 in subject 15; module 1 for subject 16) consists of FDS and FCR/U activities, and was mainly used for power grip and wrist flexion; its mean (SD) *R*-value was 0.68 (0.25). Note that these three modules were also found commonly among moderately-impaired subjects as well (see Fig. 5C).

The fourth muscle modules exhibited relatively low similarity across the unimpaired subjects, as indicated by its low *R*-values. This module (module 3 in subject 15; module 4 in subject 16) mainly consisted of HTH activity, with the mean (SD) *R*-value of 0.44 (0.18).

Overall, the similarity analysis confirmed that the scalar-product similarity values between the muscle modules of stroke survivors were lower than those of unimpaired subjects (Table 4, Table 5, Table 6). The similarity of the muscle modules were the highest among between unimpaired subjects (Table 5: mean *R* = 0.69); the similarity between unimpaired and stroke subjects was somewhat smaller (Table 6: mean *R* = 0.64); the similarity between stroke survivors was lowest of all (Table 4: mean *R* = 0.53). Similarly, the percentage of muscle module pairs with significant similarity, as determined by exceeding a critical *R-*value of 0.68 (*p = *0.05; see Methods), was largest between unimpaired subjects (13 out of 24 pairs; 54.2%), followed by between stroke and unimpaired subjects (90 out of 188 pairs; 47.9%), and smallest for similarities between stroke survivors (96 out of 269 pairs; 35.7%).

**Table 4:** Scalar-product similarity coefficient (*R*) between muscle modules: between stroke survivors.

		Group		
		S2	S3	S4
**Group**	S2	0.50 (0.48)	0.45 (0.21)	0.55 (0.30)
	S3		0.45 (0.21)	0.57 (0.18)
	S4			0.65 (0.22)

* Group S2: stroke survivors with two muscle modules (*n* = 2), group S3: stroke survivors with three muscle modules (*n* = 5), group S4: stroke survivors with four muscle modules (*n* = 7).

**Table 5:** Scalar-product similarity coefficient (*R*) between muscle modules: between unimpaired subjects.

		Group	
		U4	U5
**Group**	U4	0.70 (0.28)	0.66 (0.24)

* Group U4: unimpaired subjects with four muscle modules (*n* = 3), group U5: an unimpaired subject with five muscle modules (*n* = 1).

**Table 6:** Scalar-product similarity coefficient (*R*) between muscle modules: between stroke survivors and unimpaired subjects.

		Group		
		S2	S3	S4
**Group**	U4	0.61 (0.17)	0.61 (0.18)	0.67 (0.21)
	U5	0.68 (0.06)	0.66 (0.19)	0.62 (0.23)

* Group S2: stroke survivors with two muscle modules (*n* = 2), group S3: stroke survivors with three muscle modules (*n* = 5), group S4: stroke survivors with four muscle modules (*n* = 7); group U4: unimpaired subjects with four muscle modules (*n* = 3), group U5: an unimpaired subject with five muscle modules (*n* = 1).

**Figure 7:**
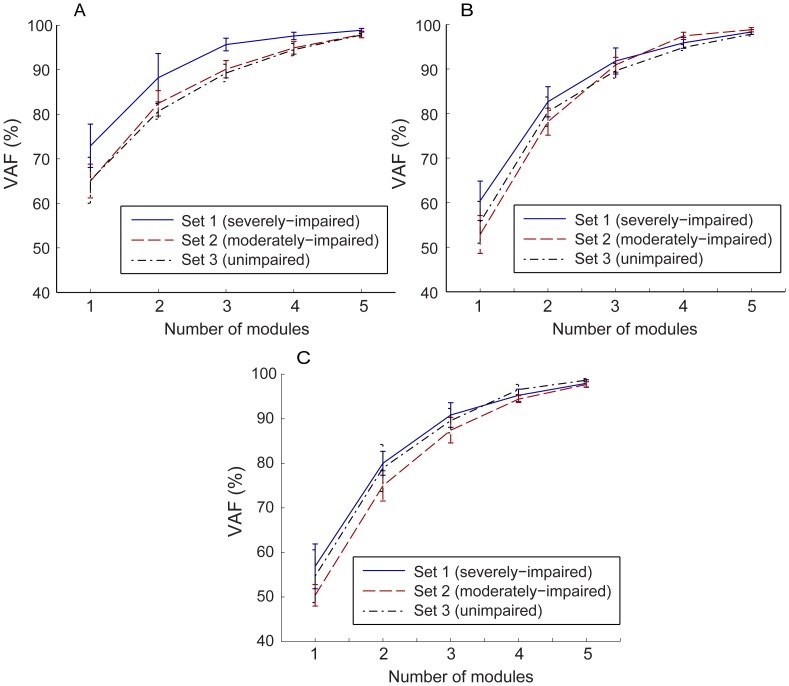
VAF by the muscle modules extracted from the three subject groups: VAF estimated from the muscle coordination patterns of (A) severely-impaired subjects, (B) moderately-impaired subjects, and (C) unimpaired subjects, when reconstructed by the three module sets.

In the cross-validation procedure, when only a small number of muscle modules (i.e., 1 or 2 modules) were used to reconstruct the muscle activation patterns, the modules of the severely-impaired group explained larger variance for all three groups (Fig. 7A–7C). When more than three modules were used in the cross-validation, there was little difference in the VAF values, and for all subject groups, all of the three VAF values constructed by three modules were significantly greater than the critical VAF value estimated from three random modules (VAF_crit_ = 87.4% at 95% confidence interval; *p*<0.05). Apparently, when more than three modules were used, the VAF value for each subject group was the highest when its own muscle modules were employed.

The reconstruction of muscle modules of patients by merging those of healthy subjects showed similar results, i.e., dissimilarity in module composition among severely-impaired subjects. The muscle modules of stroke survivors with a higher number of modules (i.e., 4 modules; all moderately-impaired subjects) were well reconstructed by merging/combining the muscle modules of unimpaired subjects (mean scalar-product similarity *R = *0.87), but the reconstruction of the modules of those with a lower number of modules (e.g., 2 or 3 modules) were not as successful (mean *R* = 0.79 for 3 modules; mean *R* = 0.76 for 2 modules; mostly severely-impaired subjects).

### Increased Co-variation of Activation Level of Muscle Pairs across Tasks

Correlation analyses of the activation level of muscle pairs revealed that the level of correlation of muscle pairs across tasks was much higher in the severely impaired group (mean *R* = 0.57; Table 7) than in the moderately impaired group (mean *R* = 0.26; Table 8). The correlation between muscle pairs across tasks was even smaller in subjects with no impairment (mean *R* = 0.17; Table 9). Furthermore, bootstrap procedures with 95% confidence intervals identified that, in severely impaired subjects, 88% of these correlation coefficients were found to be significantly different from zero, while 65% and 64% of the coefficients were statistically different from zero for moderately impaired subjects and subjects with no impairment, respectively.

**Table 7:** Co-variation matrices (correlation coefficient *r*) of activation levels of muscle pairs across tasks, averaged within three subject groups: severely-impaired subjects (CMSH 2–3).

	THE	FDI	HTH	EDC	FDS	ECR/ECU	FCR/FCU
THE	1	0.61	0.56	0.52	0.40	0.57	0.45
FDI		1	0.65	0.70	0.63	0.67	0.62
HTH			1	0.56	0.47	0.59	0.50
EDC				1	0.54	0.76	0.58
FDS					1	0.57	0.56
ECR/U						1	0.53
FCR/U							1

**Table 8:** Co-variation matrices (correlation coefficient *r*) of activation levels of muscle pairs across tasks, averaged within three subject groups: moderately-impaired subjects (CMSH 4–5).

	THE	FDI	HTH	EDC	FDS	ECR/ECU	FCR/FCU
THE	1	0.33	0.10	0.16	0.32	0.56	0.25
FDI		1	0.13	0.15	0.36	0.14	0.24
HTH			1	0.10	0.31	0.28	0.37
EDC				1	0.07	0.60	0.21
FDS					1	0.25	0.66
ECR/U						1	0.27
FCR/U							1

**Table 9:** Co-variation matrices (correlation coefficient *r*) of activation levels of muscle pairs across tasks, averaged within three subject groups: subjects with no impairment (CMSH 7).

	THE	FDI	HTH	EDC	FDS	ECR/ECU	FCR/FCU
THE	1	0.64	0.15	0.03	0.16	0.06	−0.07
FDI		1	−0.08	−0.20	0.20	−0.17	−0.08
HTH			1	0.42	0.11	0.31	0.34
EDC				1	0.25	0.57	0.23
FDS					1	0.18	0.49
ECR/U						1	−0.02
FCR/U							1

Specifically, the level of correlation was the highest in EDC-ECR/U (mean *R* = 0.76) and FDI-EDC (mean *R* = 0.70) muscle pairs for the severely impaired subject group, and FDS-FCR/U (mean *R* = 0.66) and EDC-ECR/U (mean *R* = 0.60) for the moderately impaired subject group. The muscle pairs FDI-THE (mean *R* = 0.64) and EDC-ECR/U (mean *R* = 0.57) showed the greatest correlation for the no-impairment subjects.

**Figure 8:**
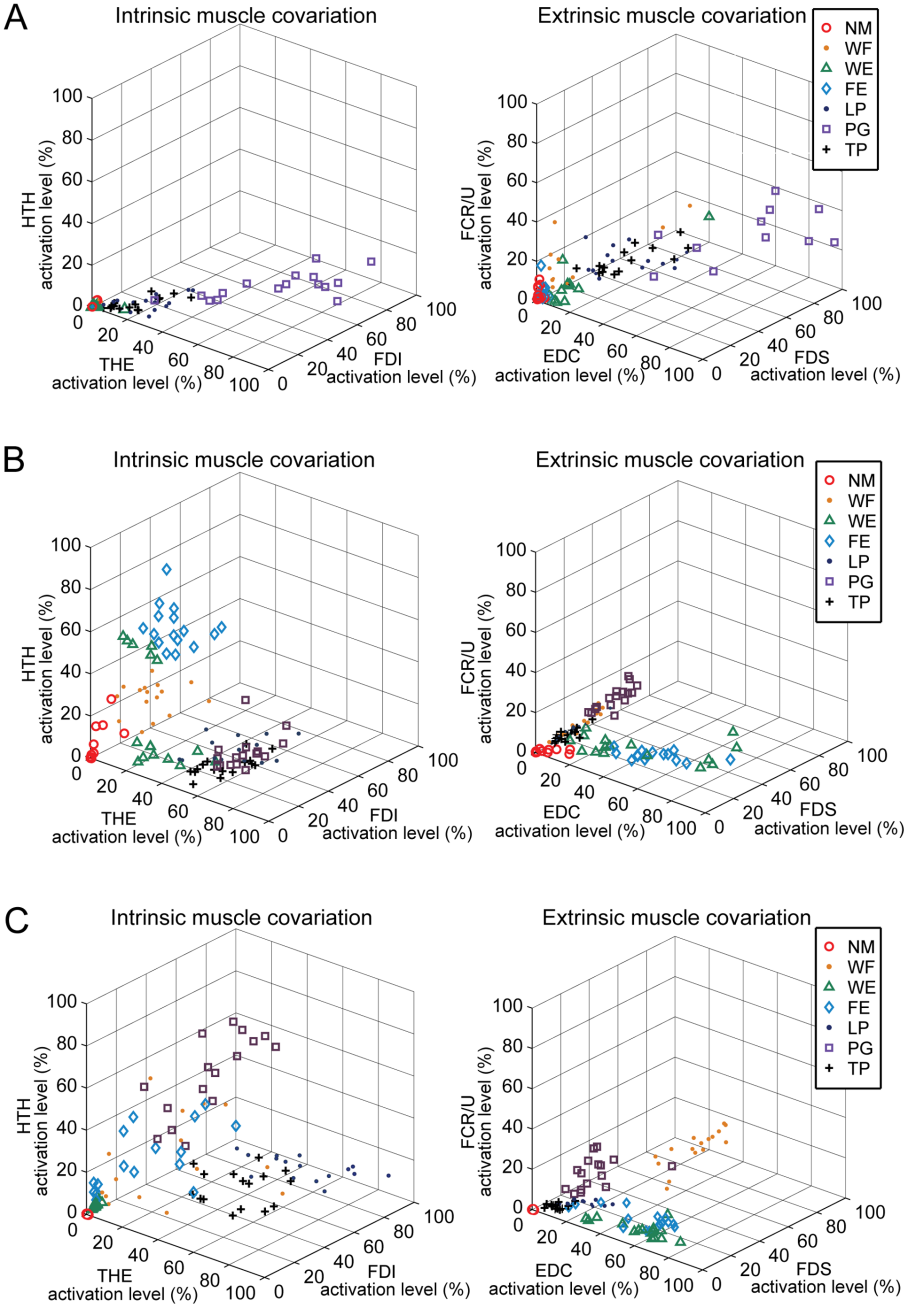
Muscle co-variation pattern across task: (A) Severely impaired subject (Subject 3) (B) Moderately impaired subject (Subject 12) (C) Subject with no impairment (Subject 16).

Correlation patterns in the muscle activation levels across task demonstrate noticeable dimensionality reduction in muscle activation patterns post-stroke, as indicated in the two subspaces of the muscle workspace (Fig. 8). For severely impaired subjects, correlation of the muscles tends to be confined within a single dimension in both subspaces (i.e., linear correlation across tasks implying simple changes in overall amplitude; Fig. 8A). Moderately impaired subjects demonstrated slightly increased ‘dimensionality’ in their muscle activation patterns when compared to severely impaired subjects, as more than a single linear relationship was observed among muscle activation levels (i.e., two linear trends coexisting in Fig. 8B). This is closer to the patterns obtained from the subjects without impairment (Fig. 8C), in which no distinct co-variation pattern was present. Note that, for severely-impaired subjects, even the within-task variability of muscle correlation was often restricted to the same linear relationship shown in the between-task variability (Fig. 8A).

## Discussion

### Reduction in the Muscle Activation Workspace Associated with Impairment Level

In theory, each of the muscle groups monitored for this study can be activated independently. Thus, the 7 muscle groups would constitute a 7-dimensional space of muscle activations which could be fully explored by the generation of appropriate neural commands. Our results, however, show a substantially diminished capacity to reach portions of this potential workspace during the six target tasks, especially in stroke survivors with more severe clinical impairment. Inter-task separation in the multi-muscle activation patterns of severely impaired stroke survivors, assessed by the off-diagonal components of their SED matrices, was much lower than that of subjects with moderate impairment, thereby indicating that the severely impaired subjects employed relatively similar muscle activation patterns across tasks. The inter-task separation values for the moderately impaired subjects were much closer in value to those obtained from the young, neurologically intact subjects.

More importantly, the motor complexity, captured by extracted modular structure from muscle coordination patterns across six tasks, was found to be significantly compromised, contributing to the reduced inter-task separation. Fewer modules were needed to reconstruct the muscle activation patterns of stroke survivors with severe hand impairment across tasks than for the patterns of subjects with moderate hand impairment. The degree of the motor complexity, quantified by the number of muscle modules employed during the target task performance, was well correlated to the degree of hand impairment, as assessed by CMSH (*R* = 0.68). For severely impaired subjects, a small number of muscle modules were extracted from the activation patterns for the six target tasks (Fig. 5), indicating a greater similarity in their muscle activation patterns across tasks. These subjects tend to employ very similar muscle activation patterns for different tasks (i.e., although the overall activation level is considerably varied with task); therefore, only two muscle modules were required to reconstruct their activation profiles for all six tasks (Fig. 5A). These subjects were unable to adapt their activation patterns to the given task requirements and, therefore, were either unable to fully complete the task or had noticeable difficulties performing these tasks. For moderately impaired subjects, in contrast, four modules were extracted from their muscle activation patterns. They were able to produce more distinct activation patterns across tasks. Note that, for the subjects without neurological impairment, four or five distinct muscle modules were extracted from the muscle activation patterns seen across the six tasks (Fig. 6); these unimpaired subjects were able to produce muscle activation patterns generally specific to each functional task of a distinct nature (although tasks of a similar nature, such as lateral pinch and tip pinch, had similar activation patterns).

The smaller number of modules corresponds to a smaller portion of the available muscle activation workspace that can be accessed by the stroke survivors. Certain regions of the workspace cannot be reached, as exemplified by examination of a three-dimensional subset (i.e., three muscles) of the overall workspace (Fig. 8). Co-variation among muscle activations in stroke subjects often restricted utilization of this three-dimensional workspace to a lower dimensional space such as a plane (Fig. 8B) or even a line (Fig. 8A). Thus, the richness of the activation patterns that could be generated was limited, thereby diminishing the functional capabilities of the hand. Note that such dimensionality reduction post-stroke, i.e., decrease in the number of muscle modules, was not observed when muscle modules were extracted from and compared between tasks of similar nature (i.e., multi-directional isometric force generation with the arm [56], [57]). However, the dimensionality reduction similar to our results, i.e., decrease in the number of muscle modules, was indeed observed in other studies involving stroke survivors during gait [53] and functional UE tasks [55]. Here, we postulate that the complexity of the target task did contribute to the reduction in the modular structure in these studies. Note that human gait generally consists of multiple stages, for which complex spatiotemporal coordination of multiple muscles is continuously required [69]. In this context, the human gait can be regarded as a successive performance of multiple sub-tasks, for which a task-specific modulation of muscle activation patterns would be crucial. The other study [55] that examined the muscle modular structure in UE musculature did record the muscle coordination patterns of stroke survivors performing various functional UE tasks [55]. Therefore, an impaired ability to modulate muscle coordination patterns across functional tasks could be one of the major factors that contribute to the functional impairment post-stroke, as examined in these studies.

### Nature of Dimensionality Reduction

Although inter-subject variability in the muscle activation patterns certainly existed in subjects with no impairment, similarities in the elemental muscle modules across subjects could be identified. The first module, containing mainly thenar and FDI activity, was largely active in the three grip tasks. The second module, consisting of EDC and ECR/U, was heavily weighted, as expected, during finger/wrist extension. The third module, involving coactivation of the wrist and finger muscles, was most prominent during power grip and wrist flexion, and the fourth module, with FCR/U and EDC activity, was employed during wrist flexion (Fig. 6). For those individuals with five modules, the first module (THE and FDI) was divided into two modules, each of which was prominent during lateral or tip pinch.

For stroke survivors who utilized four independent modules (i.e., subjects with moderate impairment; CMSH 4–5), the composition of these modules were generally similar to those of subjects with no impairment (see Fig. 5C and Table 6), which implies that these subjects did not adopt new motor control strategies, but rather were able to retain prior strategies [53]. This similarity in the muscle module composition indicates that the subjects with moderate impairment were able to access most of the muscle workspace available to unimpaired subjects for the tasks and muscle groups evaluated in this study.

In contrast, for severely impaired subjects, each muscle module contains substantially increased co-activation of multiple muscles; it was common for one module to contain more than three dominant muscle activation components (e.g., Fig. 5A and B). In addition, each module was usually activated during more than three or four tasks. Our *post-hoc* analysis showed that the muscle modules of subjects with two or three modules were not well represented by simply merging modules of healthy subjects, indicating that there might be a significant change in the muscle modular structure of the more impaired subjects. Modules for the severely impaired subjects consisted of more general, rather than task-specific, activation. Thus, the first module from the severely impaired subjects explained more of the EMG variance in the data for the other groups than even the first module for the corresponding group. Additional modules from the severely impaired group, however, accounted for less of the variance than the modules specific to the moderately impaired and unimpaired groups.

We performed a similarity analysis between the identified muscle modules among subjects who possess the same number of modules to examine commonality of the modules. As a group, the severely impaired subjects (with two or three modules), displayed significant inter-subject variability in terms of the composition of the muscle modules, suggesting that these module shapes may reflect the subject-specific impairment characteristics. For example, all muscle modules of subject 3 (CMSH 3) contain very low levels of activation of intrinsic muscles (THE and FDI), indicating that the loss of intrinsic muscle control was significant. In contrast, for the other severely impaired subject whose muscle activation patterns also can be described with two muscle modules (subject 7), one muscle module contains a high level of these intrinsic muscle activities. The inter-subject variability of muscle modules among moderately impaired subjects (and subjects with no impairment) was significantly lower, thereby suggesting that task-dependent modulation patterns of muscle activation was relatively preserved in moderately impaired subjects. Note that the muscle modules of patients with a small number of modules (i.e., 2 or 3) were not as well reconstructed by healthy modules as those of patients with four modules. Note that the similarity between muscle modules was the lowest between the stroke survivors (average *R*-value = 0.53; 35.7% of the module pairs have *R*-values higher than a chance level), and highest between the unimpaired subjects (average *R-*value = 0.68; 54% of the module pairs have *R*-values higher than a chance level).

The correlation analysis results underscored this greater coactivation among muscles in stroke survivors, especially in severely impaired subjects (Table 7). Certainly, some tasks presented in this study are similar in nature (e.g. lateral pinch and tip pinch), which might contribute to the increased level of co-variation, and some muscle pairs appear to be activated in a “synergistic” way (co-activated) more frequently than others, even in subjects with no impairment. For example, finger flexor-wrist flexor (FDS-FCR/U) and finger extensor/wrist extensor (EDC-ECR/U) pairs had some of the highest correlation coefficients. Still, the overall extent of co-variation of muscle pairs in severely impaired subjects was considerably greater than that of subjects with no impairment or of moderately impaired subjects (Table 7, Table 8, Table 9). Consequently, similar muscle activation patterns were used during attempts to perform tasks of very different natures in these subjects (e.g., Subjects 3 and 6 in Fig. 3A and B). This inability to control individual muscles/muscle groups in a task-dependent manner appears to be a significant factor contributing to functional degradation. Therefore, it appears that two distinct types of alterations in the muscle modules emerge following stroke. First, the nature of individual modules may change, with a tendency for greater co-contraction of certain muscles with greater clinical impairment, in accordance with previous studies [23], [34], [53], [67]. This may be attributable to post-lesional cortical reorganization [70]. More importantly, the number of the available modules decreases, indicating that the dimensionality of the solution space itself is reduced, which may have even greater impact on the functional degradation of the hand. The richness of the activation patterns which can be created is diminished and with it the flexibility to perform the variety of tasks performed with the hand.

### Other Impairment Characteristics

One of the primary differences in muscle activation patterns between stroke survivors and subjects with no impairment was the activation level of the intrinsic hand muscles, specifically thenar and FDI muscles, during tasks. Neurologically intact subjects generally activated their intrinsic muscles to a considerable degree (40%–50% of their maximal contraction) during manual tasks and wrist isometric contractions, but stroke survivors appeared not to utilize their intrinsic muscles to the same extent during these tasks (only approximately 20% of their maximal contraction), possibly due to diminished ability to activate these muscles effectively because of corticospinal damage. Note that muscle activation levels were normalized by the maximum values which could be generated by a given subject (see section 2.3.1). Thus, stroke survivors used even less of the available (i.e., maximum) activation of the intrinsic muscles, which is already much smaller than those of neurologically intact subjects. In absolute terms, the differences are undoubtedly much greater.

Additionally, severely impaired stroke survivors demonstrated very low activation levels of all muscles during isometric wrist flexion/extension and finger extension tasks (see Figs. 3A and B), but their muscle activation levels significantly increased (monotonically) across all muscles during all three grip tasks. Hence, muscle recruitment was easier during grasp than during hand opening. This is consistent with the relatively greater impairment of hand opening and object release as compared to grasp [19], [71]. Specifically, among the six target tasks, these subjects showed a very low activation level for all muscles during isometric wrist muscle contraction and finger extension, and all muscle modules were used to a very small degree during these tasks (i.e., the tuning curves in Fig. 5A and B). We believe that the voluntary recruitment of muscle modules responsible for wrist tasks and finger extension were more impaired in these subjects, resulting in a very low level of overall muscle activation for these tasks. Conversely, overall activation was greater for grasping, possibly due to the influence of brainstem pathways such as the reticulospinal pathway [72].

### Methodological Considerations

The analytic methods used in this study are based upon the hypothesis that humans utilize flexible combinations of muscle modules in order to produce complex motor behavior. Although a number of recent studies have provided evidence in support of this hypothesis, other studies have also suggested alternatives to the use of muscle modules, such as ‘minimum-intervention hypothesis [73]’ or ‘uncontrolled-manifold concept [74]’, as the major principles of human motor control organization. Thus, analyses of the muscle coordination patterns employing such alternative motor control principles proposed by these studies could lead to different (but also viable) explanation of the abnormalities in muscle coordination.

It should be acknowledged that only a limited number of hand muscles are considered in this study. For example, neither flexor digitorum profundus nor the interossei (except FDI) were monitored despite their involvement for most of the grip tasks tested in this study. Note, however, that the objective of this study was not to fully explain the muscle activation patterns that enable functional tasks, but to examine the task-dependent modulation patterns of selected muscles. Nevertheless, we acknowledge that the activation pattern data from these muscles excluded in this study should provide more information regarding altered motor control of stroke survivors. Additionally, the abnormalities in the multi-muscle coordination were examined during static/isometric tasks in this study. As the degree of impairment post-stroke is highly dependent upon the movement-related parameters such as velocity (e.g., spasticity; [75]), it is possible that observed impairment in the ability to modulate multi-muscle activation patterns may be further exacerbated during functional movements of the digits.

We normalized muscle activities by their maximal voluntary contraction data, therefore the activation levels reported in this study may be elevated for the stroke subjects, as full muscle activation is difficult for stroke survivors to achieve [76]. This is especially true for the hand extensor muscles, which showed increased activation levels during most grip tasks, substantially higher than any flexor muscles. Thus, the absolute degree of variability could be overestimated as noise would be amplified to a relatively greater extent due to normalization by a much smaller value, but the relative degree of variability across tasks would still be valid.

Many stroke subjects exhibited weak voluntary activations of a number of muscles, specifically finger extensor (EDC) and wrist muscles (i.e., ECR, ECU. FCR, and FCU), possibly affecting the accuracy of electrode placement. For some of the severely impaired subjects, we examined the unimpaired arm of the subject to identify the location of some muscles that did not show much visible contraction. In an effort to detect potential cross-talk between recording electrodes, the EMG signals from all target muscles were simultaneously displayed on a screen after the electrodes were placed on the upper extremity. This allowed us to visually inspect all the signals when subjects produced targeted contractions of each of the nine muscles; if any cross-talk was detected, placement of the corresponding recording electrodes was changed until perceived cross-talk was eliminated. Similar procedures were adopted in our previous studies with stroke survivors, which allowed us to attain good differentiation between the muscles (e.g., [77]).

We acknowledge that the neurologically intact subjects were younger than the stroke subjects, which could contribute to different activation patterns. For example, differences in the SED values defining the inter-task separation between moderately impaired and neurologically intact subjects may have been partly attributable to the age difference between the groups [78]–[81]. However, even if there exists an age-related change in the muscle activation patterns, such as increased co-activation of antagonist muscle, task-specific modulation of muscle activation is found to be generally unaffected by age [82]. Motor unit recruitment characteristics are also found to remain unaffected by age [83]. In fact, in our study, although the moderately impaired group (mean age = 61.0) was generally older than the severely-impaired group (mean age = 56.2), the muscle activation patterns of the moderately impaired group resulted in greater SED values than the severely impaired group, and these values were closer to those of the unimpaired subjects. Similarly, ‘older’ subjects with moderate impairment indeed employed more muscle modules than ‘younger’ subjects with severe impairment.

Lastly, potential variability in the hand and wrist postures across subjects may have contributed to the variability in the muscle activation patterns. Subjects were not asked to maintain specific finger or wrist postures, as most stroke survivors (specifically severely impaired subjects) tend to adopt subject-specific strategies to facilitate task performance (e.g., [51]). As the effects of posture on hand muscle coordination can be significant in some cases (e.g., [84]), such variation in wrist/finger posture may have increased the between-subject variability in their muscle activation patterns.

### Implications

It has long been hypothesized that cortical damage can lead to impairment in the ability to independently activate muscles, as an increased reliance on brainstem pathways (e.g. vestibulospinal or reticulospinal) can result when higher-level motor pathways (i.e., corticospinal) are compromised [85], [86]. Indeed, our recent study suggests that the common cortical descending drive to muscles of stroke survivors is diminished, resulting in much smaller EMG-EMG coherence values compared to those of subjects with no impairment [87]. The results of this study, i.e., impairment in the muscle module structure of stroke survivors, lead us to surmise that the reduction in motor complexity may arise from disruption of the descending cortical pathways. Indeed, recent studies suggest that brainstem pathways, particularly the reticulospinal tract, may play a larger role in control of the arm [88], [89] and hand [70] post-stroke. The altered muscle modules and manipulation of these modules may arise from the influence of these brainstem pathways. For example, reticulospinal excitation produces a flexor bias in the upper extremity [90], similar to what is observed post-stroke. This study effectively links the reduction in the voluntary motor activation space to the degree of clinical impairment. The reduction in the degrees-of-freedom of muscle coordination following stroke, along with other peripheral changes such as muscle spasticity and/or weakness, would significantly affect the functionality of the hand of those affected. Similar to the findings of this study, the loss of independent muscle control was found to be prominent in severely impaired subjects, but not in subjects with moderate or mild impairment in arm function [91]. Thus, restoration of the motor complexity should be a primary target for therapy, focusing on full exploration of the muscle activation workspace. This could include creation of patterns not typically associated with functional tasks. Indeed, in a previous study we guided stroke survivors to produce fingertip forces in a variety of directions [92]. This generalized training led to improved production of normally directed pinching forces. Similarly, a training study with spinalized rats showed that training of backwards or sideways locomotion led to greater improvement of forward stepping than actual practice of the forward stepping task [93]. Thus, workspace exploration may be more conducive to rehabilitation than repetitive practice of a specific task.

## References

[1] Roger VL, Go AS, Lloyd-Jones DM, Benjamin EJ, Berry JD, et al. (2012) Heart disease and stroke statistics–2012 update: a report from the American Heart Association. Circulation 125; e2– e220. 10.1161/CIR.0b013e31823ac046 PMC4440543 22179539

[2] Trombly CA (1989) Stroke. In: Trombly CA, Ed *Occupational therapy for physical dysfunction*. Baltimore, MD: Williams and Wilkins: 454–71.

[3] Bohannon RW, Smith MB (1987) Assessment of strength deficits in eight paretic upper extremity muscle groups of stroke patients with hemiplegia. Phys Ther 67: 522–525. 3562543 10.1093/ptj/67.4.522

[4] Cruz EG, Waldinger HC, Kamper DG (2005) Kinetic and kinematic workspace of the index finger following stroke. Brain 128: 1112–1121. 15743873 10.1093/brain/awh432

[5] Triandafilou K, Fischer HC, Towles JD, Kamper DG, Rymer WZ (2011) Diminished capacity to modulate motor activation patterns according to task contributes to thumb deficits following stroke. J Neurophysiol 106: 1644–1651. 21753022 10.1152/jn.00936.2010

[6] Sukai TM, Ellis MD, Dewald JPA (2007) Shoulder abduction-induced reductions in reaching work area following hemiparetic stroke: neuroscientific implications. Exp Brain Res 183: 215–223. 17634933 10.1007/s00221-007-1029-6 PMC2827935

[7] Beebe JA, Lang CE (2009) Active range of motion predicts upper extremity function 3 months after stroke. Stroke 40: 1772–1779. 19265051 10.1161/STROKEAHA.108.536763 PMC2718540

[8] Kamper DG, Cruz EG, Siegel MP (2003) Stereotypical fingertip trajectories during grasp, J Neurophysiol. 90: 3702–3710. 10.1152/jn.00546.2003 12954607

[9] Beer RF, Dewald JPA, Dawson ML, Rymer WZ (2004) Target-dependent differences between free and constrained arm movements in chronic hemiparesis. Exp Brain Res 156: 458–470. 14968276 10.1007/s00221-003-1807-8

[10] Seo NJ, Rymer WZ, Kamper DG (2010) Altered digit force direction during pinch grip following stroke, Exp Brain Res. 202: 891–901. 10.1007/s00221-010-2193-7 20186401

[11] O’Dwyer NJ, Ada L, Neilson PD (1996) Spasticity and muscle contracture following stroke, Brain. 119: 1737–1749. 10.1093/brain/119.5.1737 8931594

[12] Given JD, Dewald JPA, Rymer WZ (1995) Joint dependent passive stiffness in paretic and contralateral limbs of spastic patients with hemiparetic stroke. J Neurol Neurosurg Psychiatry 59: 271–279. 7673955 10.1136/jnnp.59.3.271 PMC486028

[13] Dattola R, Girlanda P, Vita G, Santoro M, Roberto ML, et al (1993) Muscle Rearrangement in Patients with Hemiparesis after Stroke: An Electrophysiological and Morphological Study, Eur Neurol. 33: 109–114. 10.1159/000116915 8467816

[14] McPherson JG, Ellis MD, Heckman CJ, Dewald JPA (2008) Evidence for increased activation of persistent inward currents in individuals with chronic hemiparetic stroke, J Neurophysiol. 100: 3236–324. 10.1152/jn.90563.2008 PMC2604864 18829849

[15] Lance JW (1980) Symposium synopsis. In: Feldman RG, Young RR, Koella WP, Eds. Spasticity: disordered motor control. Miami: Symposia Specialists: 485–94.

[16] Schmit BD, Dewald JPA, Rymer WZ (2000) Stretch reflex adaptation in elbow flexors during repeated passive movements in unilateral brain-injured patients. Arch Phys Med Rehabil 81: 269–278. 10724069 10.1016/s0003-9993(00)90070-4

[17] Sommerfeld DK, Eek EU, Svensson AK, Holmqvist LW, von Arbin MH (2004) Spasticity after stroke: its occurrence and association with motor impairments and activity limitations, Stroke. 35: 134–139. 10.1161/01.STR.0000105386.05173.5E 14684785

[18] Canning CG, Ada L, O’Dwyer NJ (2000) Abnormal muscle activation characteristics associated with loss of dexterity after stroke. J Neurol Sci 176: 45–56. 10865092 10.1016/s0022-510x(00)00305-1

[19] Kamper DG, Rymer WZ (2001) Impairment of voluntary control of finger motion following stroke: role of inappropriate muscle coactivation. Muscle Nerve 24: 673–681. 11317278 10.1002/mus.1054

[20] Seo NJ, Rymer WZ, Kamper DG (2009) Delays in Grip Initiation and Termination in Persons with Stroke: Effects of Arm Support and Active Muscle Stretch Exercise, J Neurophysiol. 101: 3108–3115. 10.1152/jn.91108.2008 19357330

[21] Gemperline JJ, Allen S, Walk D, Rymer WZ (1995) Characteristics of motor unit discharge in subjects with hemiparesis, Muscle Nerve. 18: 1101–1114. 10.1002/mus.880181006 7659104

[22] Zhou P, Suresh NL, Rymer WZ (2007) Model based sensitivity analysis of EMG - force relation with respect to motor unit properties: applications to muscle paresis in stroke, Ann Biomed Eng. 35: 1521–1531. 10.1007/s10439-007-9329-3 17530407

[23] Gowland C, deBruin H, Basmajian JV, Plews N, Burcea I (1992) Agonist and antagonist activity during voluntary upper-limb movement in patients with stroke, Phys Ther. 72: 624–633. 10.1093/ptj/72.9.624 1508970

[24] Patten C, Lexell J, Brown HE (2004) Weakness and strength training in persons with poststroke hemiplegia: Rationale, method, and efficacy, J Rehabil Res Dev. 41: 293–312. 10.1682/jrrd.2004.03.0293 15543447

[25] Klein CS, Brooks D, Richardson D, McIlroy WE, Bayley MT (2010) Voluntary activation failure contributes more to plantar flexor weakness than antagonist coactivation and muscle atrophy in chronic stroke survivors, J App Physiol. 109: 1337–1346. 10.1152/japplphysiol.00804.2009 20724561

[26] Brunnstrom S (1970) Movement therapy in hemiplegia: a neurophysiological approach. New York: Harper and Row.

[27] Beer RF, Given JD, Dewald JP (1999) Task-dependent weakness at the elbow in patients with hemiparesis, Arch Phys Med Rehabil. 80: 766–772. 10.1016/s0003-9993(99)90225-3 10414760

[28] Cirstea MC, Levin MF (2000) Compensatory strategies for reaching in stroke, Brain. 123: 940–953. 10.1093/brain/123.5.940 10775539

[29] Dewald JPA, Sheshadri V, Dawson ML, Beer RF (2001) Upper limb discoordination in hemiparetic stroke: implication for neurorehabilitation, Top Stroke Rehabil. 8: 1–12. 10.1310/WA7K-NGDF-NHKK-JAGD 14523747

[30] Lum PS, Burgar CG, Shor PC (2003) Evidence for strength imbalances as a significant contributor to abnormal synergies in hemiparetic subjects, Muscle Nerve. 27: 211–221. 10.1002/mus.10305 12548529

[31] Ellis MD, Holubar BG, Acosta AM, Beer RF, Dewald JPA (2005) Modifiability of abnormal isometric elbow and shoulder joint torque coupling after stroke, Muscle Nerve. 32: 170–178. 10.1002/mus.20343 PMC2847894 15880629

[32] Welmer AK, Holmqvist LW, Sommerfeld DK (2006) Hemiplegic limb synergies in stroke patients, Am J Phys Med Rehabil. 85: 112–119. 10.1097/01.phm.0000197587.78140.17 16428901

[33] Dipietro L, Krebs HI, Fasoli SE, Volpe BT, Stein J, et al (2007) Changing motor synergies in chronic stroke, J Neurophysiol. 98: 757–768. 10.1152/jn.01295.2006 17553941

[34] Dewald JPA, Pope PS, Given JD, Buchanan TS, Rymer WZ (1995) Abnormal muscle coactivation patterns during isometric torque generation at the elbow and shoulder in hemiparetic subjects, Brain. 118: 495–510. 10.1093/brain/118.2.495 7735890

[35] Iwamuro BT, Cruz EG, Connelly LL, Fischer HC, Kamper DG (2008) Effect of a gravity-compensating orthosis on reaching after stroke: evaluation of the therapy assistant WREX, Arch Phys Med Rehabil. 89: 2121–2128. 10.1016/j.apmr.2008.04.022 18996241

[36] Schieber MH, Santello M (2004) Hand function: peripheral and central constraints on performance. J App Physiol 96: 2293–2300. 10.1152/japplphysiol.01063.2003 15133016

[37] An KN, Chao EY, Cooney WP, Linscheid RL (1985) Forces in the normal and abnormal hand, J Orthop Res. 3: 202–211. 10.1002/jor.1100030210 3998897

[38] Cole KJ, Abbs JH (1986) Coordination of three-joint digit movements for rapid finger-thumb grasp, J Neurophysiol. 55: 1407–1423. 10.1152/jn.1986.55.6.1407 3734863

[39] Dennerlein JT, Diao E, Mote CD Jr, Rempel DM (1998) Tensions of the flexor digitorum superficialis are higher than a current model predicts, J Biomech. 31: 295–301. 10.1016/s0021-9290(98)00006-2 9672082

[40] Valero-Cuevas FJ, Zajac FE, Burgar CG (1998) Large index-fingertip forces are produced by subject-independent patterns of muscle excitation, J Biomech. 31: 693–703. 10.1016/s0021-9290(98)00082-7 9796669

[41] Lee SW, Chen H, Towles JD, Kamper DG (2008) Estimation of the effective static moment arms of the tendons in the index finger extensor mechanism, J Biomech. 41: 1467–1573. 10.1016/j.jbiomech.2008.02.008 18387615

[42] Napier JR (1956) The prehensile movements of the human hand, J Bone Joint Surg. 38B: 902–913. 10.1302/0301-620X.38B4.902 13376678

[43] Elliot JM, Connolly KJ (1984) A classification of manipulative hand movements, Dev Med Child Neurol. 26: 283–296. 10.1111/j.1469-8749.1984.tb04445.x 6734945

[44] Maier MA, Hepp-Reymond MC (1995) EMG activation patterns during force production in precision grip. I. Contribution of 15 finger muscles to isometric force, Exp Brain Res 103: 108–122. 7615027 10.1007/BF00241969

[45] Valero-Cuevas FJ (2000) Predictive modulation of muscle coordination pattern magnitude scales fingertip force magnitude over the voluntary range, J Neurophysiol. 83: 1469–1479. 10.1152/jn.2000.83.3.1469 10712473

[46] Kutch JJ, Kuo AD, Bloch AM, Rymer WZ (2008) Endpoint force fluctuations reveal flexible rather than synergistic patterns of muscle cooperation, J Neurophysiol. 100: 2455–2471. 10.1152/jn.90274.2008 PMC2585402 18799603

[47] Rearick MP, Casares A, Santello M (2003) Task-dependent modulation of multi-digit force coordination patterns, J Neurophysiol. 89: 1317–26. 10.1152/jn.00581.2002 12626614

[48] Weiss EJ, Flanders M (2004) Muscular and postural synergies of the human hand, J Neurophysiol. 92: 523–535. 10.1152/jn.01265.2003 14973321

[49] Raghavan P, Krakauer JW, Gordon AM (2006) Impaired anticipatory control of fingertip forces in patients with a pure motor or sensorimotor lacunar syndrome, Brain. 129: 1415–1425. 10.1093/brain/awl070 PMC2093998 16597653

[50] Takahashi CD, Reinkensmeyer DJ (2003) Hemiparetic stroke impairs anticipatory control of arm movement, Exp Brain Res. 149: 131–140. 10.1007/s00221-002-1340-1 12610680

[51] Raghavan P, Santello M, Gorden AM, Krakauer JW (2010) Compensatory motor control after stroke: an alternative joint strategy for object-dependent shaping of hand posture, J Neurophysiol. 103: 3034–3043. 10.1152/jn.00936.2009 PMC2888236 20457866

[52] Holdefer RN, Miller LE (2002) Primary motor cortical neurons encode functional muscle synergies, Exp Brain Res. 146: 233–243. 10.1007/s00221-002-1166-x 12195525

[53] Clark DJ, Ting LH, Zajac FE, Neptune RR, Kautz SA (2010) Merging of healthy motor modules predicts reduced locomotor performance and muscle coordination complexity post-stroke, J Neurophysiol. 103: 844–857. 10.1152/jn.00825.2009 PMC2822696 20007501

[54] Cheung VCK, Piron L, Agostin M, Silvoni S, Turolla A, et al (2009) Stability of muscle synergies for voluntary actions after cortical stroke in humans, PNAS. 106: 19563–19568. 10.1073/pnas.0910114106 PMC2780765 19880747

[55] Cheung VCK, Turolla A, Agostini M, Silvoni S, Bennis C, et al (2012) Muscle synergy patterns as physiological markers of motor cortical damage, PNAS. 109: 14652–14656. 10.1073/pnas.1212056109 PMC3437897 22908288

[56] Roh J, Rymer WZ, Perreault EJ, Yoo SB, Beer RF (2012) Alterations in upper limb muscle synergy structure in chronic stroke survivors, J Neurophysiol. 109: 768–781. 10.1152/jn.00670.2012 PMC3567389 23155178

[57] Roh J, Rymer WZ, Beer BF (2012) Robustness of muscle synergies underlying three-dimensional force generation at the hand in healthy humans, J Neurophysiol. 107: 2123–2142. 10.1152/jn.00173.2011 PMC3331600 22279190

[58] Gowland CA (1990) Staging motor impairment after stroke, Stroke 21 (suppl. II): 19–21. 2399544

[59] Johnston JA, Bobichb LR, Santello M (2010) Coordination of intrinsic and extrinsic hand muscle activity as a function of wrist joint angle during two-digit grasping, Neurosci Letters. 474: 104–108. 10.1016/j.neulet.2010.03.017 PMC2855501 20227463

[60] Lindeman RH, Merenda PF, Gold RZ (1979) Introduction to Bivariate and Multivariate Analyses, Scott Foresman & Co, Glenview, IL.

[61] Lee SW, Wilson K, Lock BA, Kamper DG (2011) Subject-specific myoelectric pattern classification for stroke survivors, IEEE Trans Neural Syst Rehabil Eng. 19: 558–566. 10.1109/TNSRE.2010.2079334 PMC4010155 20876030

[62] Tresch MC, Saltiel P, Bizzi E (1999) The construction of movement by the spinal cord, Nat Neurosci. 2: 162–167. 10.1038/5721 10195201

[63] Ting LH, Macpherson JM (2005) A limited set of muscle synergies for force control during a postural task, J Neurophysiol. 93: 609–613. 10.1152/jn.00681.2004 15342720

[64] d’Avella A, Bizzi E (2004) Shared and specific muscle synergies in natural motor behaviors, Proc Natl Acad Sci USA. 102: 3076–3081. 10.1073/pnas.0500199102 PMC549495 15708969

[65] Torres-Oviedo G, Ting LH (2007) Muscle synergies characterizing human postural responses, J Neurophysiol. 98: 2144–2156. 10.1152/jn.01360.2006 17652413

[66] Lee DD, Seung HS (1999) Learning the parts of objects by non-negative matrix factorization, Nature. 401: 276–308. 10.1038/44565 10548103

[67] Bowden MG, Clark DJ, Kautz SA (2010) Evaluation of abnormal synergy patterns poststroke: relationship of the Fugl-Meyer assessment to hemiparetic locomotion, Neurorehabil Neural Repair. 24: 328–337. 10.1177/1545968309343215 PMC4434590 19794132

[68] Frere J, Hug F (2012) Between-subject variability of muscle synergies during a complex motor skill, Front Comput Neurosci. 6: 99. 10.3389/fncom.2012.00099 PMC3531715 23293599

[69] Zajac FE, Neptune RR, Kautz SA (2002) Biomechanics and muscle coordination of human walking. Part I: Introduction to concepts, power transfer, dynamics and simulations, Gait Posture 16: 215–232. 12443946 10.1016/s0966-6362(02)00068-1

[70] Traversa R, Cicinelli P, Bassi A, Rossini PM, Bernardi G (1997) Mapping of motor cortical reorganization after stroke. A brain stimulation study with focal magnetic pulses, Stroke 28: 110–117. 8996498 10.1161/01.str.28.1.110

[71] Lang CE, Schieber MH (2004) Reduced muscle selectivity during individuated finger movements in humans after damage to the motor cortex or corticospinal tract, J Neurophysiol. 91: 1722–1733. 10.1152/jn.00805.2003 14668295

[72] Baker SN (2011) The primate reticulospinal tract, hand function and functional recovery, J Physiol 589; 5603–5612. 10.1113/jphysiol.2011.215160 PMC3249036 21878519

[73] Valero-Cuevas FJ, Venkadesan M, Todorov E (2009) Structured variability of muscle activations supports the minimum intervention principle of motor control, J Neurophysiol. 102: 59–68. 10.1152/jn.90324.2008 PMC2712269 19369362

[74] Scholz JP, Schoner G (1999) The uncontrolled manifold concept: identifying control variables for a functional task, Exp Brain Res. 126: 289–306. 10.1007/s002210050738 10382616

[75] Kamper DG, Rymer WZ (2000) Quantitative features of the stretch response of extrinsic finger muscles in hemiparetic stroke, Muscle Nerve. 23: 954–961. 10.1002/(sici)1097-4598(200006)23:6<954::aid-mus17>3.0.co;2-0 10842274

[76] Patten C, Srisethnil J, Asakawa D, Wright GA, Gold G (2002) Imaging activation impairment in post-stroke hemiparesis, Proc Intl Soc Magn Reson Med 10^th^ Annu Meet, Honolulu, HI.

[77] Hoffmann G, Kamper DG, Kahn JH, Rymer WZ, Schmit BD (2009) Modulation of stretch reflexes of the finger flexors by sensory feedback from the proximal upper limb poststroke, J Neurophysiol. 102: 1420–1429. 10.1152/jn.90950.2008 PMC2746792 19571191

[78] Galganski ME, Fuglevand AJ, Enoka RM (1993) Reduced control of motor output in a human hand muscle of elderly subjects during submaximal contractions. J Neurophysiol 69: 2108–2115. 8350134 10.1152/jn.1993.69.6.2108

[79] Spiegel KM, Stratton J, Burke JR, Glendinning DS, Enoka RM (1996) The influence of age on the assessment ofz motor unit activation in a human hand muscle, Exp Physiol. 81: 805–819. 10.1113/expphysiol.1996.sp003978 8889479

[80] Laidlaw DH, Bilodeau M, Enoka RM (2000) Steadiness is reduced and motor unit discharge is more variable in old adults. Muscle Nerve 23: 600–612. 10716772 10.1002/(sici)1097-4598(200004)23:4<600::aid-mus20>3.0.co;2-d

[81] Ranganathan VK, Siemionow V, Sahgal V, Yue GH (2001) Effects of aging on hand function, J Am Geriatr Soc. 49: 1478–1484. 10.1046/j.1532-5415.2001.4911240.x 11890586

[82] Burnett RA, Laidlaw DH, Enoka RM (2000) Coactivation of the antagonist muscle does not covary with steadiness in old adults, J Appl Physiol. 89: 61–71. 10.1152/jappl.2000.89.1.61 10904036

[83] Jesunathadas M, Marmon AR, Gibb JM, Enoka RM (2010) Recruitment and derecruitment characteristics of motor units in a hand muscle of young and old adults, J Appl Physiol. 108: 1659–1667. 10.1152/japplphysiol.00807.2009 PMC2886691 20339011

[84] Johnston JA, Bobichb LR, Santello M (2010) Coordination of intrinsic and extrinsic hand muscle activity as a function of wrist joint angle during two-digit grasping, Neurosci Letters. 474: 104–108. 10.1016/j.neulet.2010.03.017 PMC2855501 20227463

[85] Lawrence DG, Kuypers HG (1968) The functional organization of the motor system in the monkey, II. The effects of lesions of the descending brain-stem pathways, Brain 91: 15–36. 4966860 10.1093/brain/91.1.15

[86] Bourbonnais D, Vanden Noven S, Carey KM, Rymer WZ (1989) Abnormal spatial patterns of elbow muscle activation in hemiparetic human subject, Brain. 112: 85–102. 10.1093/brain/112.1.85 2917281

[87] Conrad MO, Kamper DG, Lee SW (2011) Common cortical drive to hand muscles altered following stroke, Society for Neuroscience Annual Meeting, Washington, DC.

[88] Ellis MD, Acosta AM, Yao J, Dewald JPA (2007) Position-dependent torque coupling and associated muscle activation in the hemiparetic upper extremity, Exp Brain Res. 176: 594–602. 10.1007/s00221-006-0637-x PMC2827933 16924488

[89] Schwerin S, Dewald JPA, Haztl M, Jovanovich S, Nickeas M, et al. (2008) Ipsilateral versus contralateral cortical motor projections to a shoulder adductor in chronic hemiparetic stroke: implications for the expression of arm synergies, Exp Brain Res 185; 509–519. 10.1007/s00221-007-1169-8 PMC2831614 17989973

[90] Davidson AG, Bufford JA (2006) Bilateral actions of the reticulospinal tract on arm and shoulder muscles in the monkey: stimulus triggered averaging, Exp Brain Res. 173: 25–39. 10.1007/s00221-006-0374-1 16506008

[91] Reinkensmeyer DJ, Cole AM, Kahn LE, Kamper DG (2002) Directional control of reaching is preserved following mild/moderate stroke and stochastically constrained following severe stroke, Exp Brain Res. 143: 525–530. 10.1007/s00221-002-1055-3 11914800

[92] Seo NJ, Fischer HW, Bogey RO, Rymer WZ, Kamper DG (2011) Use of visual force feedback to improve digit force direction during pinch grip in persons with stroke: A pilot study, Arch Phys Med Rehabil. 92: 24–30. 10.1016/j.apmr.2010.08.016 21092931

[93] Shah PK, Gerasimenko Y, Shyu A, Lavrov I, Zhong H, et al (2012) Variability in step training enhances locomotor recovery after a spinal cord injury. Eur J Neurosci 36: 2054–2062. 22591277 10.1111/j.1460-9568.2012.08106.x PMC3389255

